# Association Between Maternal Factors and Risk of Congenital Heart Disease in Offspring: A Systematic Review and Meta-Analysis

**DOI:** 10.1007/s10995-022-03538-8

**Published:** 2022-11-07

**Authors:** Lina Wu, Na Li, Yong Liu

**Affiliations:** Department of Laboratory Medicine, Shengjing Hospital of China Medical University, Shenyang, China

**Keywords:** Congenital heart defects, Meta-analysis, Offspring, Pregnancy, Systematic review

## Abstract

**Introduction:**

This study aimed to summarize the evidence describing the relationship between maternal factors during gestation and risk of congenital heart disease (CHD) in offspring.

**Methods:**

PubMed, EMBASE, and the Cochrane Library were searched for potentially relevant reports from inception to May 2021. Pooled odds ratios (ORs) with 95% confidence intervals (CIs) calculated by the random-effects model were used to evaluate the association between maternal factors and CHD risk.

**Results:**

There was a significant association between CHD risk and obesity in pregnancy (OR 1.29, 95% CI 1.22–1.37; *P* < 0.001), smoking in pregnancy (OR 1.16, 95% CI 1.07–1.25; *P* < 0.001), maternal diabetes (OR 2.65, 95% CI 2.20–3.19; *P* < 0.001), and exposure of pregnant women to organic solvents (OR 1.82, 95% CI 1.23–2.70; *P* = 0.003). No correlations were revealed between CHD susceptibility and advanced maternal age (OR 1.04, 95% CI 0.96–1.12; *P* = 0.328), underweight (OR 1.02, 95% CI 0.96–1.08; *P* = 0.519), alcohol intake in pregnancy (OR 1.08, 95% CI 0.95–1.22; *P* = 0.251), coffee intake (OR 1.18, 95% CI 0.97–1.44; *P* = 0.105), and exposure to irradiation (OR 1.80, 95% CI 0.85–3.80; *P* = 0.125).

**Discussion:**

Maternal factors including maternal obesity, smoking in pregnancy, maternal diabetes and exposure to organic solvents might predispose the offspring to CHD risk.

**Supplementary Information:**

The online version contains supplementary material available at 10.1007/s10995-022-03538-8.

## Significance

*What is already known on this subject?* Several maternal factors including overweight and obesity were associated with CHD progression in children. Maternal underweight was found not to be associated with increased susceptibility to CHD in offspring.

*What this study adds?* Other maternal factors including smoking, diabetes and exposure to organic solvents were significantly associated with an elevated risk of CHD in children. In addition, maternal alcohol or coffee intake, and exposure to irradiation showed no associations with CHD risk in offspring. Furthermore, these associations could be influenced by study design, reported outcomes and models adopted to adjust for confounders.

## Introduction

CHD represents the most common malformation diagnosed in newborns globally, and is mainly characterized by incomplete cardiac development from 1 to 6 weeks of pregnancy. The prevalence of CHD was found to be nearly 4–5/1000 live births, with the highest rate reported in Asia (9.3/1000 live births) and the lowest rate in Africa (1.9/1000 live births) (Hoffman & Kaplan, 2002; van der Linde et al., 2011). Moreover, with the passage of time, the prevalence of CHD showed an “S”-shaped graph, with 0.6/1000 and 9.1/1000 live births recorded in 1930 and 2011, respectively (van der Linde et al., 2011). Most patients are diagnosed with severe CHD through cardiac catheterization. Although several medical and surgical strategies have been developed to improve the survival rate of infants with CHD, not all neonatal patients can be successfully treated with surgery. The treatment effects are related to social, economic and personal factors (Kirklin et al., 1990, 1992; Murphy et al., 1993). Moreover, the long-term prognosis of CHD infants undergoing surgeries is not clearly determined (Pacifico et al., 1990). Therefore, the importance and potential impact of maternal factors on primary prevention of CHD disease in offspring should be determined.

Multiple systematic reviews and meta-analyses have been performed to assess the influence of maternal factors on CHD progression in offspring. Sun et al. meta-analyzed 19 case–control and 4 cohort studies, and the results demonstrated that maternal alcohol intake was not significantly associated with CHD in offspring (Sun et al., 2015). Hoang et al. reported that pre-gestational diabetes had a significant linkage with all CHD phenotypes (Hoang et al., 2017). Zhu et al. meta-analyzed 13 case–control and 4 cohort studies, and found that maternal overweight and obesity, rather than maternal underweight, were associated with increased susceptibility to CHD in offspring (Zhu et al., 2018). However, several other maternal factors potentially influencing CHD risk in offspring, including maternal age, smoking history, coffee intake, irradiation and exposure to organic solvents, were not addressed in the aforementioned studies. Therefore, this meta-analysis aimed to comprehensively examine the available reports to clarify the relationship between multiple maternal-associated factors and CHD risk in children.

## Methods

### Search Strategy and Study Selection

The current systematic review and meta-analysis was carried out in accordance with the Preferred Reporting Items for Systematic Reviews (PRISMA) guideline. PubMed, EMBASE, and the Cochrane Library were systematically queried for potentially relevant reports from inception to May 2021, with “maternal” AND “congenital heart disease” AND “infant” OR “newborn” OR “offspring” as core search terms. Observational studies evaluating the associations between maternal factors and CHD risk in offspring were eligible for screening, with no restrictions on language and publication status. Then, a thorough review of the reference lists of relevant reports was performed to manually identify additional eligible studies.

Based on eligibility criteria, two investigators carried out the search in an independent manner, and a third investigator was involved in case of a disagreement between them. The inclusion criteria were: (1) all participants being pregnant women, with the number of CHD cases in children reported; (2) two or more studies investigating the same maternal factors including age, body mass index (BMI), alcohol intake, smoking history, diabetes, coffee intake, irradiation, and exposure to organic solvents; (3) outcomes including the risk of CHD, and atrial (ASD) and ventricular (VSD) septal defects in children; (4) reporting effect estimates and corresponding 95% confidence intervals (CIs) or raw data from which the effect estimates and 95% CIs could be calculated and combined. The exclusion criteria were: (1) review; (2) animal studies; (3) studies not reporting the estimates of the influence of maternal factors on offspring CHD. All included studies in this meta-analysis were approved by the appropriate ethics committee and were performed in accordance with the 1964 Declaration of Helsinki and its later amendments. All patients gave their informed consents prior to enrollment in all included prospective studies.

### Data Collection and Study Quality Evaluation

The data collected included the first author’s surname, publication year, trial design, country, CHD case and non-CHD case numbers, maternal factors, outcomes, and confounding factors. The quality of retrieved studies were assessed using the Newcastle–Ottawa Scale (NOS), which encompassed four selection, one comparability and three outcome subscales. A star rating system ranging between 0 and 9 was employed for scoring observational studies (Wells et al., 2009). Study quality was independently assessed by two authors, and any discrepancies were adjudicated by a third investigator.

### Statistical Analysis

The associations between maternal factors and the risk of CHD in offspring were assigned as binary data, and effect estimates with 95% CIs were obtained from each individual study. Summary results from individual studies of maternal factors reported in multiple categories were assessed by the fixed-effects model. Then, a random-effects model was used for summarizing pooled odds ratios (ORs) and 95% CIs to determine whether there was an association between maternal factor and the risk of CHD in offspring (Ades et al., 2005; DerSimonian & Laird, 1986). Heterogeneity across included trials was evaluated by the *I*^2^ and Q statistics, with *I*^2^ > 50.0% or *P* < 0.10 indicating significant heterogeneity (Deeks et al., 2008; Higgins et al., 2003). The robustness of the overall conclusions was assessed by a sensitivity analysis that sequentially excluded individual studies (Tobias, 1999). Subgroup analysis with more than five studies was performed based on study design, reported outcomes and adjustment model. Publication bias was assessed through qualitative (funnel plot) and quantitative [Egger’s and Begg’s tests (Begg & Mazumdar, 1994; Egger et al., 1997)] measures. Two-sided *P* < 0.05 indicated statistical significance. Statistical analysis was performed with Stata 10.0 (Stata Corporation, USA).

## Results

### Included Studies

**Fig. 1:**
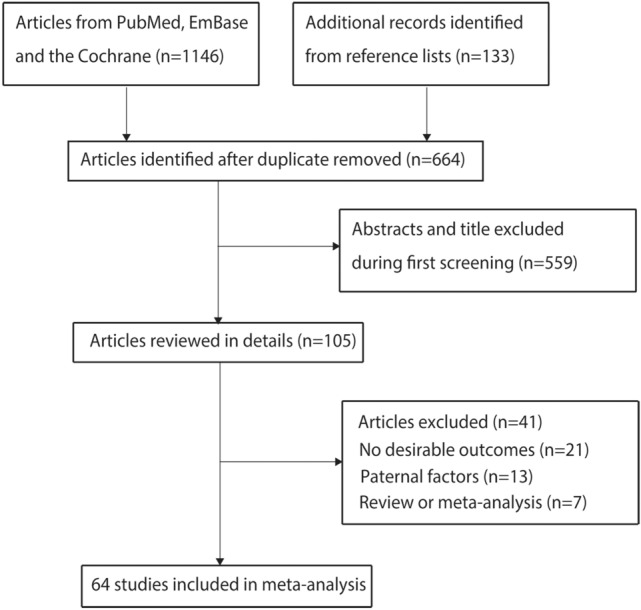
Flow diagram of the literature search and study selection process

The initial literature search yielded 1146 hits, of which 482 were excluded due to duplication. Then, 559 reports were further excluded due to irrelevance. The remaining 105 reports underwent full-text evaluation, and 41 were excluded for not reporting desirable outcomes (*n* = 21), assessing paternal factors (*n* = 13) and being a review or meta-analysis (*n* = 7). Finally, 64 observational studies were included (Bassili et al., 2000; Bean et al., 2011; Bell et al., 2012; Botto et al., 2000; Carmichael et al., 2003; Cedergren et al., 2002; Correa et al., 2012; Cresci et al., 2011; Eidem et al., 2010; Erickson, 1991; Ewing et al., 1997; Fixler & Threlkeld, 1998; Gilboa et al., 2010; Grewal et al., 2008; Hobbs et al., 2010; Janssen et al., 1996; Källén, 1999; Karatza et al., 2011; Kuciene & Dulskiene, 2009; Loffredo et al., 2001; Malik et al., 2008; Martínez-Frías et al., 2004; Martínez-Frías et al., 2005; Mateja et al., 2012; McDonald et al., 1992; Mills et al., 2010; Nielsen et al., 2005; Oddy et al., 2009; Peticca et al., 2009; Rankin et al., 2010; Sharpe et al., 2005; Shaw & Carmichael, 2008; Sheffield et al., 2002; Smedts et al., 2009; Smedts et al., 2012; Steinberger et al., 2002; Strandberg-Larsen et al., 2011; Tikkanen & Heinonen, 1991; Torfs & Christianson, 1999; van Beynum et al., 2010; van Driel et al., 2008; Waller et al., 2007; Wasserman et al., 1996; Watkins & Botto, 2001; Watkins et al., 2003; Williams et al., 2004; Woods & Raju, 2001). Reviewing their reference lists yielded 133 studies, all of which were enclosed in the initial electronic search results. The detailed study selection process was presented in Fig. 1.

### Study Characteristics

Of the 64 included studies, 46 were designed as case–control trials, while the remaining 18 adopted a cohort design. These studies assessed a total of 182,290 CHD cases in offspring. The baseline features of the included studies were presented in Table 1. Twenty-nine studies were carried out in the USA, 24 were conducted in European countries, and the remaining 11were performed in Canada, Australia, China, Egypt and Iran. Data on maternal factors influencing the risks of child ASD and VSD were available in 15 and 20 studies, respectively. Based on NOS evaluation, the rating system assigned 8 stars to 11 studies, 7 stars to 23 studies, 6 stars to 17 studies, and 5 stars to the remaining studies.

**Table 1:** Baseline characteristics of included studies

Study	Study design	Country	Cases/controls (CHD/non-CHD number)	Reported factors	Outcomes	Adjusted factors	Study quality
Erickson (1991)	CC	US	327/2889 (study 1); 179/2489 (study 2)	Diabetes	VSD; ASD	Raw data	5
Tikkanen and Heinonen (1991)	CC	Finland	573/1055	Alcohol; organic solvents; age	CHD	Alcohol, organic solvents at work, maternal age	6
McDonald et al. (1992)	Cohort	Canada	318/89,317	Smoking; alcohol; coffee	CHD	Education, ethnicity, maternal age, smoking, alcohol, and coffee	7
Wasserman et al., (1996	CC	US	207/481	Smoking	CHD	Maternal vitamin use, alcohol use, and gravidity	6
Janssen et al. (1996)	CC	US	10,379/8926	Diabetes	CHD	Maternal age, race, smoking status and year of child's birth	7
Ewing et al. (1997)	CC	US	491/3549	Smoking; alcohol; Age	VSD	Raw data	6
Fixler and Threlkeld (1998)	CC	US	89/82	Smoking; alcohol; coffee	CHD	Raw data	7
Torfs and Christianson (1999)	CC	US	385/302	Smoking; alcohol; coffee	VSD; ASD	Maternal race and age	7
Källén (1999)	Cohort	Sweden	3384/1,413,811	Smoking	CHD	Year of birth, maternal age, parity, and educational level	6
Botto et al. (2000)	CC	US	958/3029	Smoking; alcohol; Age	CHD	Raw data	7
Bassili et al. (2000)	CC	Egypt	894/894	Age, diabetes, irradiation	CHD; VSD	Paternal age, birth order, positive consanguinity, residence, positive family history, maternal and paternal occupation, psychotropic drugs, female sex hormones	8
Woods and Raju (2001)	Cohort	US	260/18,076	Smoking	CHD	Maternal age, diabetes, birth weight, gestational age, race	7
Loffredo et al. (2001)	CC	US	4390/3572	Diabetes	CHD	Maternal age, adiposity index, subfertility, hypertension, months of prenatal care, previous miscarriage, previous birth	7
Watkins and Botto (2001)	CC	US	851/2767	BMI	CHD; VSD; ASD	Race, birth period, age, education, alcohol use, smoking, chronic illness, and vitamin use. Reference category	7
Sheffield et al. (2002)	Cohort	US	214/145,196	Diabetes	CHD	Raw data	6
Steinberger et al. (2002)	CC	US	55/3572	Smoking; alcohol; smoking; age; organic solvents	CHD	Raw data	7
Cedergren et al. (2002)	CC	Sweden	277/554	Age, smoking, alcohol, BMI	CHD	Year of birth and parity	7
Carmichael et al. (2003)	CC	US	207/481	Alcohol	CHD	Maternal cigarette smoking, intake of multivitamin/mineral supplements containing folic acid, race-ethnicity and education level	7
Watkins et al. (2003)	CC	US	195/330	BMI	CHD; ASD; VSD	Raw data	6
Martinez-Frias et al. (2004)	CC	Spain	1607/1596	Alcohol	CHD	Raw data	5
Williams et al. (2004)	CC	US	122/3029	Alcohol; smoking	VSD	Raw data	7
Sharpe et al. (2005)	Cohort	Australia	2418/282,260	Diabetes	CHD	Maternal age, ethnicity, or other demographic factors	8
Nielsen et al. (2005)	CC	Denmark	4479/38,151	Diabetes	CHD	Maternal age, birth order, and use of antipsychotic drugs during pregnancy	6
Martinez-Frias et al. (2005)	CC	Spain	49/230	BMI	CHD	Maternal age, educational level and use of alcohol and/or illicit drugs	6
Waller et al. (2007)	CC	US	4128/4065	BMI	CHD	Maternal age, ethnicity, education, parity, smoking in the month prior to conception, and supplemental folic acid intake in the month prior to conception	8
Van Driel et al. (2008)	CC	The Netherlands	231/315	Alcohol; smoking	CHD	Raw data	5
Grewal et al. (2008)	CC	US	323/700	Alcohol; smoking	CHD	Raw data	6
Malik et al. (2008)	CC	US	3067/3947	Age, BMI, alcohol, and smoking	CHD; ASD; VSD	Infant gender, maternal age, race, BMI, drinking, folic acid intake, dietary folate intake, caffeine intake, family history of heart defect, and residence of mothers	8
Shaw and Carmichael (2008)	CC	US	659/700	BMI	CHD	Race/ethnicity, education, vitamin use, total energy intake, maternal height, and dietary folate intake	5
Peticca et al. (2009)	Cohort	Canada	274/53,851	Diabetes	CHD	Raw data	5
Smedts et al. (2009)	CC	The Netherlands	276/324	Alcohol, smoking	CHD	Raw data	5
Kuciene and Dulskiene (2009)	CC	Lithuania	187/643	Age, smoking, alcohol	CHD	Raw data	6
Oddy et al. (2009)	CC	Australia	111/418	BMI	CHD	Marital status, maternal age, maternal education and periconceptional folic acid supplementation	6
Eidem et al. (2010)	Cohort	Norway	3330/350,961	Diabetes	CHD	Parity and maternal age	6
Hobbs et al. (2010)	CC	US	572/363	Smoking, alcohol, BMI, age	CHD	Raw data	5
van Beynum et al. (2010)	CC	The Netherlands	611/2401	Age, BMI, smoking, alcohol	CHD	Raw data	5
Gilboa et al. (2010)	CC	US	6440/5673	Smoking, diabetes, BMI	CHD; ASD; VSD	Maternal age, race-ethnicity, education, hypertension, parity, smoking in the month prior to conception or the first month of pregnancy, and folic acid supplement use in the month prior to conception or the first month of pregnancy by conditional logistic regression grouping on study center	7
Mills et al. (2010)	CC	US	7392/56,304	Age, smoking, alcohol, BMI	CHD; ASD; VSD	Maternal age, education, race, smoking, and payment method for health care	8
Rankin et al. (2010)	Cohort	UK	270/41,013	BMI	CHD	Maternal age, ethnicity, pre-gestational diabetes, cigarette smoking status and index of multiple deprivation	6
Cresci et al. (2011)	CC	Italy	330/330	Alcohol, smoking, irradiation, organic solvents	CHD	Raw data	6
Karatza et al. (2011)	CC	Greece	157/208	Smoking, age, diabetes	CHD	Raw data	7
Bean et al. (2011)	CC	US	566/552	Age, alcohol, smoking	CHD; ASD; VSD	Raw data	5
Strandberg-Larsen et al. (2011)	Cohort	Denmark	477/80,346	Alcohol	ASD; VSD	Maternal age, parity, smoking, household occupational status, and time to pregnancy	8
Smedts et al. (2012)	CC	The Netherlands	261/325	BMI, smoking	CHD	Raw data	5
Correa et al. (2012)	CC	US	5386/4764	Diabetes	CHD; ASD; VSD	Maternal age, race, and ethnicity, entry into prenatal care, pre-pregnancy BMI, parity, and household income	7
Bell et al. (2012)	Cohort	UK	1306/401,149	Diabetes	CHD; VSD	Maternal age at delivery, gestational age at booking, preconception folic acid, nephropathy diagnosed pre-pregnancy, retinopathy diagnosed pre-pregnancy, fetal sex, parity, pre-pregnancy care, IMD, smoking during pregnancy	6
Mateja et al. (2012)	CC	US	237/948	Age, diabetes, BMI, alcohol, smoking	CHD	Any binge drinking and smoking interaction term, maternal age, maternal race, maternal ethnicity, maternal marital status, insurance, and stress	7
Liu et al. (2013)	Cohort	Canada	26,488/2,278,838	Age, alcohol, smoking, obesity, diabetes	CHD	Maternal age, infant sex, parity, rural residence, and region and year of birth	8
Madsen et al. (2013)	CC	US	11,263/140,470	BMI	CHD; ASD; VSD	Gestational diabetes	7
Cresci et al. (2011)	CC	Italy	190/190	Smoking, irradiation, organic solvents	CHD	Raw data	5
O’Leary et al. (2013)	Cohort	Australia	674/85,229	Alcohol	VSD; ASD	Maternal age, ethnicity, year of birth	8
Vereczkey et al. (2014)	CC	Hungary	1659/38,151	Diabetes	VSD; ASD	Maternal age, birth order, pregnancy order, low SES	6
Ghaderian et al. (2014)	CC	Iran	164/158	BMI	CHD	Raw data	5
Brite et al. (2014)	Cohort	US	1388/121,815	BMI	CHD; ASD; VSD	Site, age, race, insurance, maternal smoking	7
Tang et al., (2015a; 2015b)	CC	US	569/1644	Alcohol; smoking; BMI	CHD	Raw data	5
Chou et al. (2016)	Cohort	China	27,240/1,387,650	Diabetes	CHD	Maternal nationality, parental age, level of parental education, birth order, single v. multiple births, sex of the infant, infant’s year of birth, urbanization of the birth site, and maternal smoking and alcohol consumption	7
Best and Rankin (2016)	Cohort	UK	4024/499,826	Age	CHD	Year of delivery	6
Øyen et al. (2016)	Cohort	Denmark	16,325/2,025,727	Diabetes	CHD; VSD; ASD	Year of birth, maternal age at birth, and birth order	8
Li et al. (2016)	Cohort	Sweden	1499/748,951	Age, smoking, BMI, diabetes	CHD	Age, sex, family income, marital status, country of birth, education attainment, Urban/Rural status, age at child birth, socio-economic status, smoking, BMI, diabetes, alcohol, hypertension, chronic lower respiratory disease, CHD	7
Liu et al. (2018)	CC	China	1023/732	Age, smoking, alcohol, organic solvents	CHD; VSD; ASD	Ethnic, age, education level, living location, population property, household income, smoking, alcohol, organic solvents, pesticide exposure, living in newly renovated room, residential proximity to a main road < 50 m, occupation, parity, previous pregnancies with still birth, threatened abortion	7
Dolk et al. (2020)	CC	UK	242/966	Age, diabetes, smoking, alcohol, and BMI	CHD	Maternal age, previous pregnancy, maternal education, socioeconomic deprivation of the area of residence, dietary class, BMI category, self-reported folic acid supplementation, smoking, antidepressant prescription in first trimester, pregnancy stress, and multiple stressors	8
Zhao et al. (2020)	CC	China	620/620	Diabetes	CHD	Maternal age, education level, BMI, family income, residence, abnormal pregnancy history, family history, medical history, lifestyle and habits, history of exposure to environmental hazardous substances, and medicine history during pregnancy	7
Wu et al. (2020)	Cohort	USA	18,484/29,211,974	Diabetes	CHD	Maternal age, race/ethnicity, maternal education levels, marital status, parity, smoking before pregnancy, smoking during pregnancy, timing of initiation of prenatal care, prepregnancy BMI, infant sex, and prepregnancy hypertension	8
Fazekas-Pongor et al. (2021)	CC	Hungary	577/1731	BMI, diabetes, smoking, alcohol, and organic solvents	CHD	Family history of congenital anomalies, paternal age, paternal education, and paternal smoking	7

*ASD* atrial septal defect, *BMI* body mass index, *CC* case–control study, *CHD* congenital heart defects, *VSD* ventricular septal defect

### Maternal Age

**Fig. 2:**
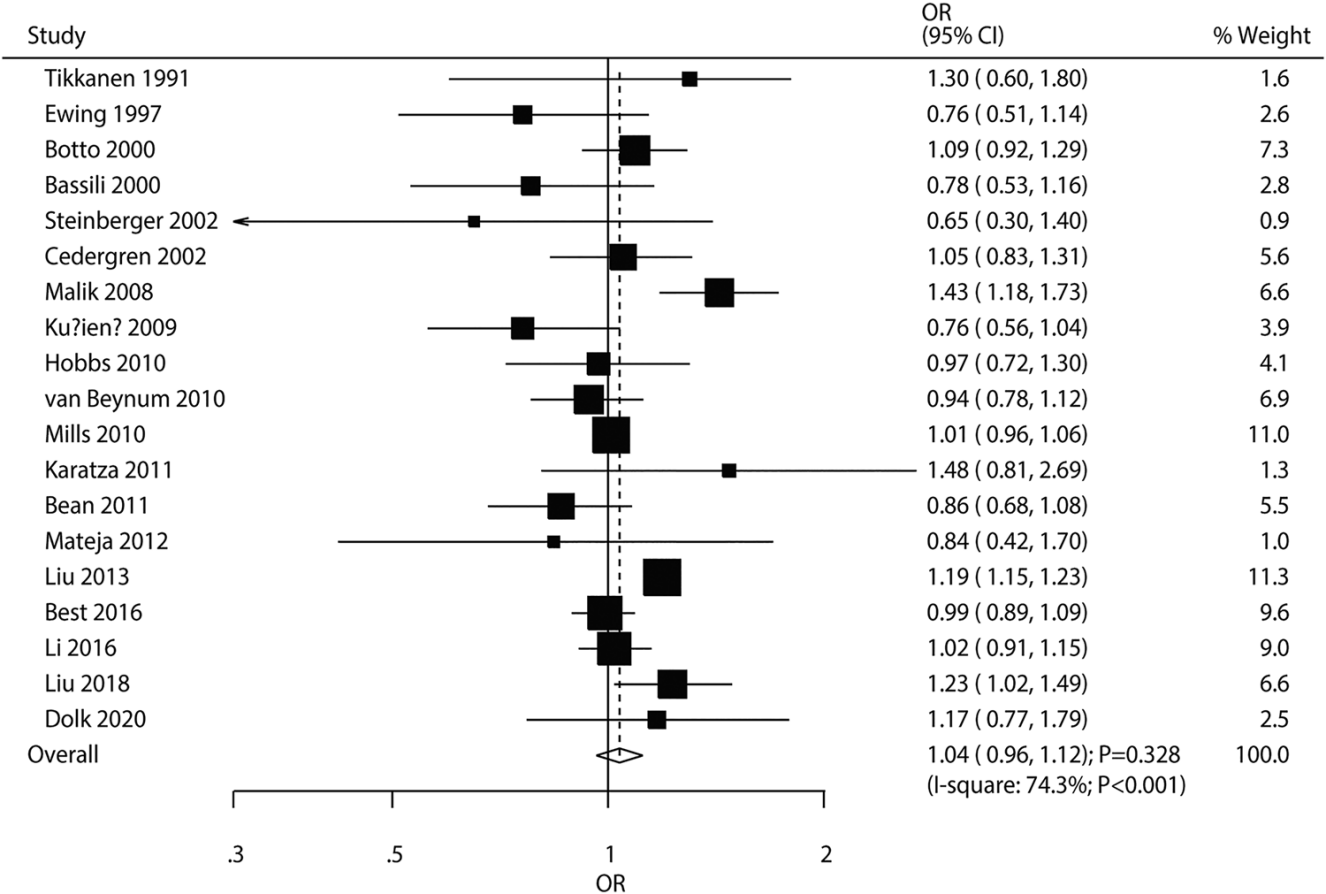
Association of maternal age with the risk of CHD in offspring

Nineteen studies assessed the influence of maternal age on CHD risk in offspring. This parameter was not significantly associated with subsequent risk of CHD in children (OR 1.04, 95% CI 0.96–1.12; *P* = 0.328; Fig. 2). Significant heterogeneity among trials was observed (*I*^2^ = 74.3%; *P* < 0.001). The above conclusion was unaltered after sequential exclusion of individual studies (Supplemental 1). Subgroup analysis revealed no significant correlation of maternal age with CHD risk in various subsets. After adjustment for potential confounding factors, advanced maternal age was shown to be associated with increased CHD risk in offspring (OR 1.09, 95% CI 1.00–1.19; *P* = 0.041; Table 2). No significant publication bias (*P*_Egger,_ = 0.092, *P*_Begg_ = 0.624; Supplemental 2) was revealed.

**Table 2:** Subgroup analyses

Outcome	Factor	Group	OR and 95% CI	*P* value	Heterogeneity (%)	*P* value for heterogeneity
Age	Study design	Case–control	1.02 (0.94–1.12)	0.601	51.7	0.009
		Cohort	1.07 (0.94–1.23)	0.322	87.7	< 0.001
	Reported outcomes	ASD	1.09 (0.78–1.53)	0.599	82.7	0.016
		VSD	0.92 (0.71–1.18)	0.508	65.8	0.032
	Adjusted results	Yes	1.09 (1.00–1.19)	0.041	79.6	< 0.001
		No	0.93 (0.83–1.05)	0.251	25.9	0.223
Obesity	Study design	Case–control	1.27 (1.19–1.35)	< 0.001	41.5	0.031
		Cohort	1.36 (1.23–1.50)	< 0.001	29.8	0.233
	Reported outcomes	ASD	1.27 (1.15–1.41)	< 0.001	38.5	0.135
		VSD	1.09 (0.98–1.20)	0.096	41.0	0.117
	Adjusted results	Yes	1.27 (1.19–1.35)	< 0.001	54.1	0.005
		No	1.44 (1.26–1.65)	< 0.001	0.0	0.615
Underweight	Study design	Case–control	1.01 (0.96–1.07)	0.669	0.0	0.687
		Cohort	1.10 (0.86–1.39)	0.458	19.8	0.288
	Reported outcomes	ASD	1.05 (0.85–1.30)	0.666	36.1	0.181
		VSD	1.01 (0.90–1.13)	0.913	0.0	0.916
	Adjusted results	Yes	1.02 (0.96–1.07)	0.601	0.0	0.742
		No	1.06 (0.80–1.40)	0.673	24.9	0.256
Alcohol	Study design	Case–control	0.99 (0.93–1.06)	0.846	16.3	0.229
		Cohort	1.31 (0.99–1.72)	0.055	91.6	< 0.001
	Reported outcomes	ASD	1.20 (0.71–2.03)	0.502	91.7	< 0.001
		VSD	1.18 (0.92–1.52)	0.184	82.7	< 0.001
	Adjusted results	Yes	1.16 (0.96–1.41)	0.124	90.9	< 0.001
		No	0.97 (0.91–1.05)	0.477	0.0	0.513
Smoking	Study design	Case–control	1.17 (1.06–1.29)	0.001	73.1	< 0.001
		Cohort	1.08 (0.98–1.20)	0.126	50.3	0.090
	Reported outcomes	ASD	1.43 (1.00–2.05)	0.053	79.3	0.001
		VSD	1.26 (1.03–1.54)	0.023	64.9	0.009
	Adjusted results	Yes	1.16 (1.06–1.27)	0.001	73.3	< 0.001
		No	1.15 (0.99–1.34)	0.062	70.3	< 0.001
Diabetes	Study design	Case–control	2.36 (1.71–3.25)	< 0.001	80.2	< 0.001
		Cohort	2.98 (2.41–3.70)	< 0.001	95.1	< 0.001
	Reported outcomes	ASD	3.20 (1.98–5.16)	< 0.001	74.2	0.004
		VSD	3.18 (2.37–4.27)	< 0.001	69.0	0.004
	Adjusted results	Yes	3.01 (2.46–3.68)	< 0.001	94.4	< 0.001
		No	1.63 (1.22–2.19)	0.001	20.7	0.266

### Maternal BMI

**Fig. 3:**
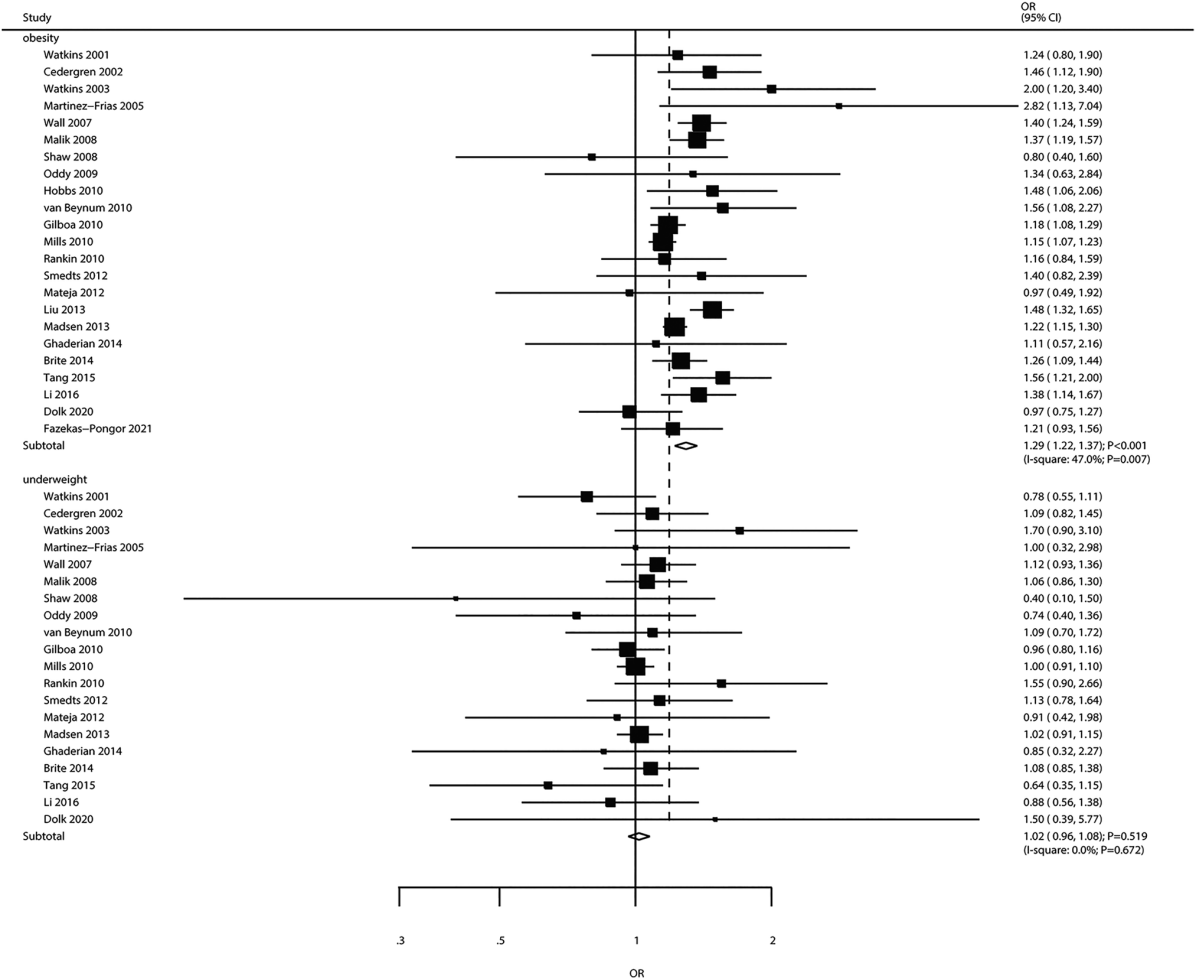
Associations of maternal obesity and underweight with the risk of CHD in offspring

Twenty-three studies assessed the influence of maternal obesity on offspring CHD risk. Maternal obesity was shown to be associated with elevated CHD risk (OR 1.29, 95% CI 1.22–1.37; *P* < 0.001; Fig. 3). There was moderate heterogeneity among trials (*I*^2^ = 47.0%; *P* = 0.007). According to sensitivity analysis, the conclusion remained unaltered after excluding individual studies (Supplemental 1). In subgroup analysis, maternal obesity was correlated with elevated CHD risk but not VSD risk (Table 2). There was no significant publication bias (*P*_Egger,_ = 0.143, *P*_Begg_ = 0.958; Supplemental 2).

There were 20 reports assessing the relationship between maternal underweight and the incidence of CHD in offspring. Maternal underweight was not significantly linked with CHD risk (OR 1.02, 95% CI 0.96–1.08; *P* = 0.519; Fig. 3). No significant heterogeneity across the studies was found (*I*^2^ = 0.0%; *P* = 0.672). The conclusion remained stable after exclusion of any given trial (Supplemental 1). In subgroup analysis, all subsets had findings consistent with the overall analysis, suggesting no significant correlation of maternal underweight with CHD risk (Table 2). There was no publication bias (*P*_Egger_ = 0.766, *P*_Begg_ = 0.871; Supplemental 2).

### Maternal Alcohol Intake

**Fig. 4:**
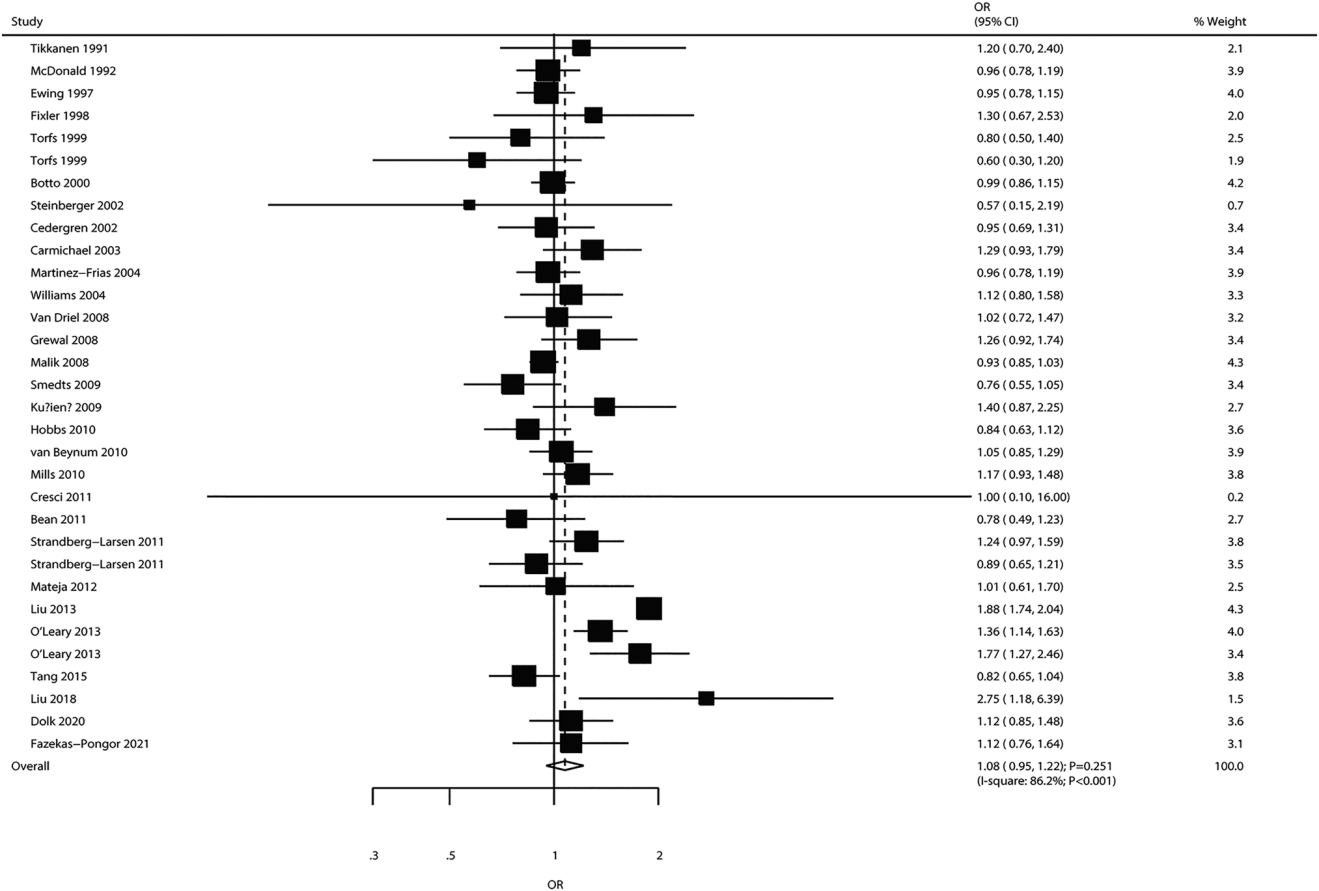
Association of maternal alcohol intake with the risk of CHD in offspring

The pooled analysis of 29 studies (32 cohorts) evaluating maternal alcohol intake suggested that this parameter was not significantly associated with CHD risk in offspring (OR 1.08, 95% CI 0.95–1.22; *P* = 0.251; Fig. 4). Although significant heterogeneity was detected among trials (*I*^2^ = 86.2%; *P* < 0.001), this conclusion remained unchanged after individual studies were sequentially excluded (Supplemental 1). Subgroup analysis showed consistent findings in various subsets. However, maternal alcohol intake might be linked with an increased risk of CHD when pooling only cohort trials (OR 1.31, 95% CI 0.99–1.72; *P* = 0.055; Table 2). No significant publication bias was detected (*P*_Egger_ = 0.053, *P*_Begg_ = 0.105; Supplemental 2).

### Maternal Smoking

**Fig. 5:**
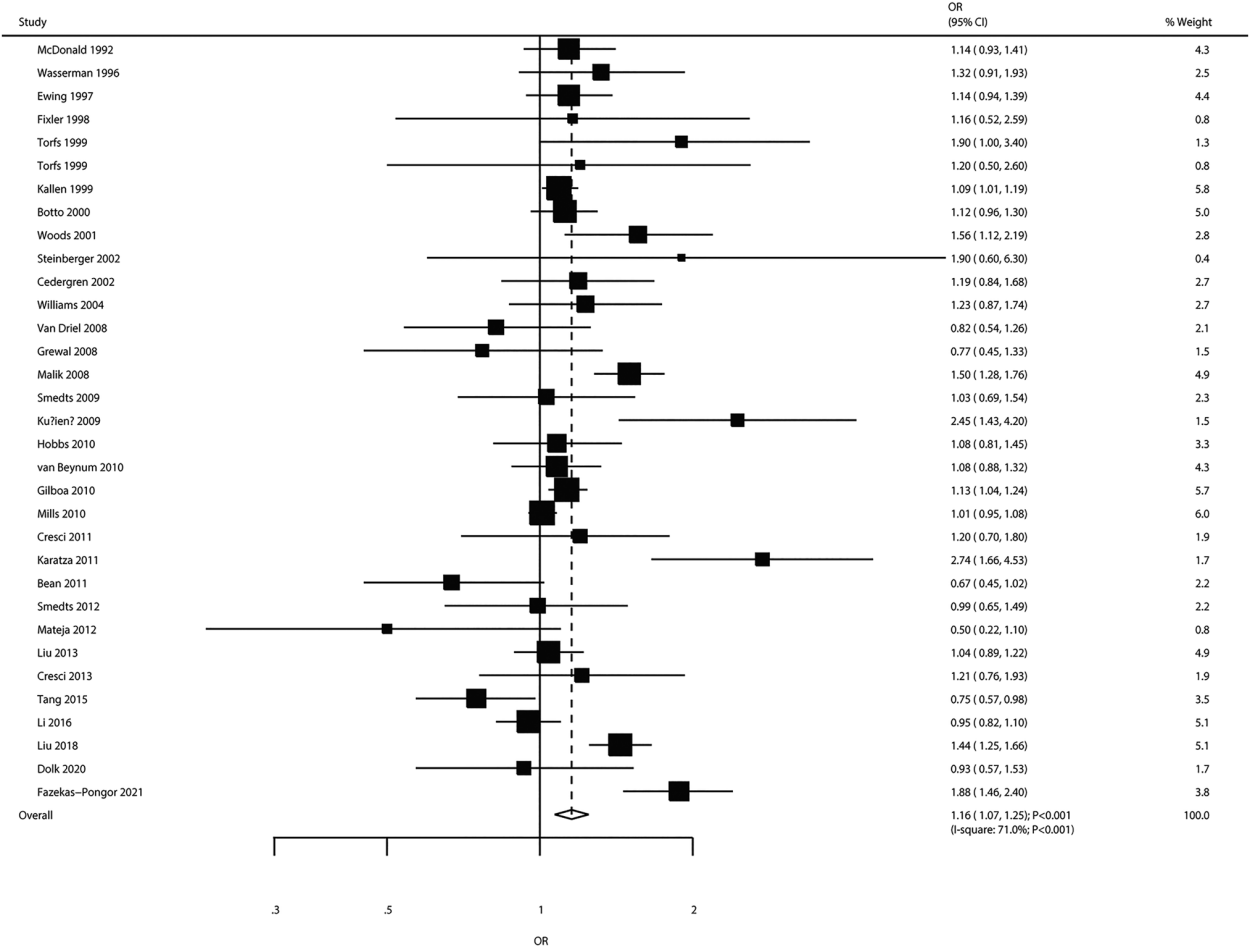
Association of maternal smoking with the risk of CHD in offspring

The effect of maternal smoking was assessed in 32 studies (33 cohorts). The pooled results showed a significant association between maternal smoking and the development of CHD in offspring (OR 1.16, 95% CI 1.07–1.25; *P* < 0.001; Fig. 5). Despite significant heterogeneity across studies (*I*^2^ = 71.0%; *P* < 0.001), the above conclusion remained unaffected by the exclusion of any particular study (Supplemental 1). In subgroup analysis, significantly increased risk was detected mainly by pooling case–control studies (OR 1.17, 95% CI 1.06–1.29; *P* = 0.001), studies reporting the VSD outcome (OR 1.26, 95% CI 1.03–1.54; *P* = 0.023), and studies adjusting for potential confounding factors (OR 1.16, 95% CI 1.06–1.27; *P* = 0.001). There was no significant publication bias (*P*_Egger_ = 0.248, *P*_Begg_ = 0.710; Supplemental 2).

### Maternal Diabetes

**Fig. 6:**
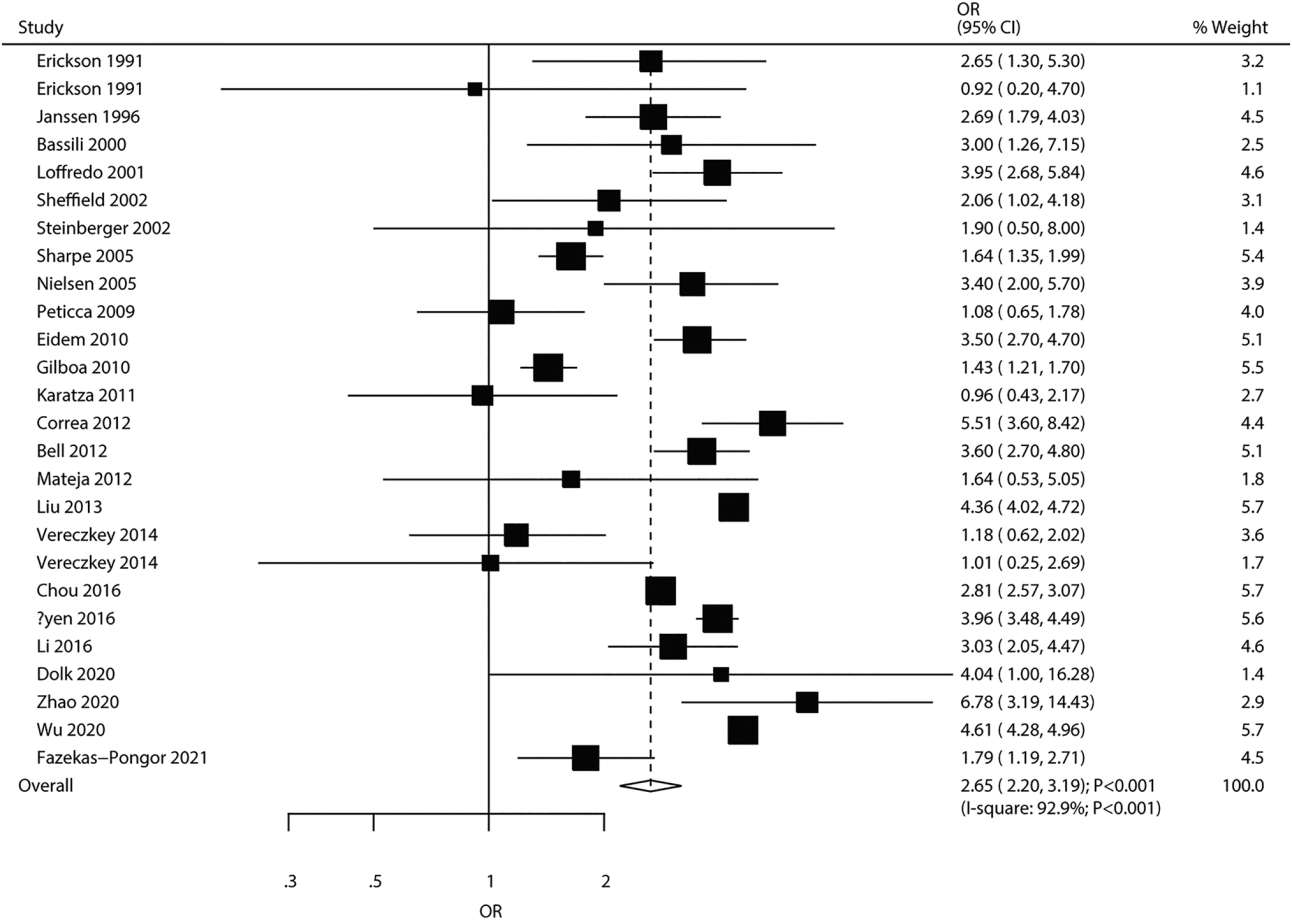
Association of maternal diabetes with the risk of CHD in offspring

The pooled results of 24 studies (26 cohorts) suggested that maternal diabetes had a significant correlation with elevated CHD risk in offspring (OR 2.65, 95% CI 2.20–3.19; *P* < 0.001; Fig. 6). There was significant heterogeneity among trials (*I*^2^ = 92.9%, *P* < 0.001). The above conclusion was robust and unaltered upon sequential exclusion of individual studies (Supplemental 1). Subgroup analysis revealed that maternal diabetes was related to increased risk of CHD in various subsets (Table 2). There was potential significant publication bias (*P*_Egger_ = 0.039, *P*_Begg_ = 0.378; Supplemental 2).

### Coffee, Irradiation, and Exposure to Organic Solvents

**Fig. 7:**
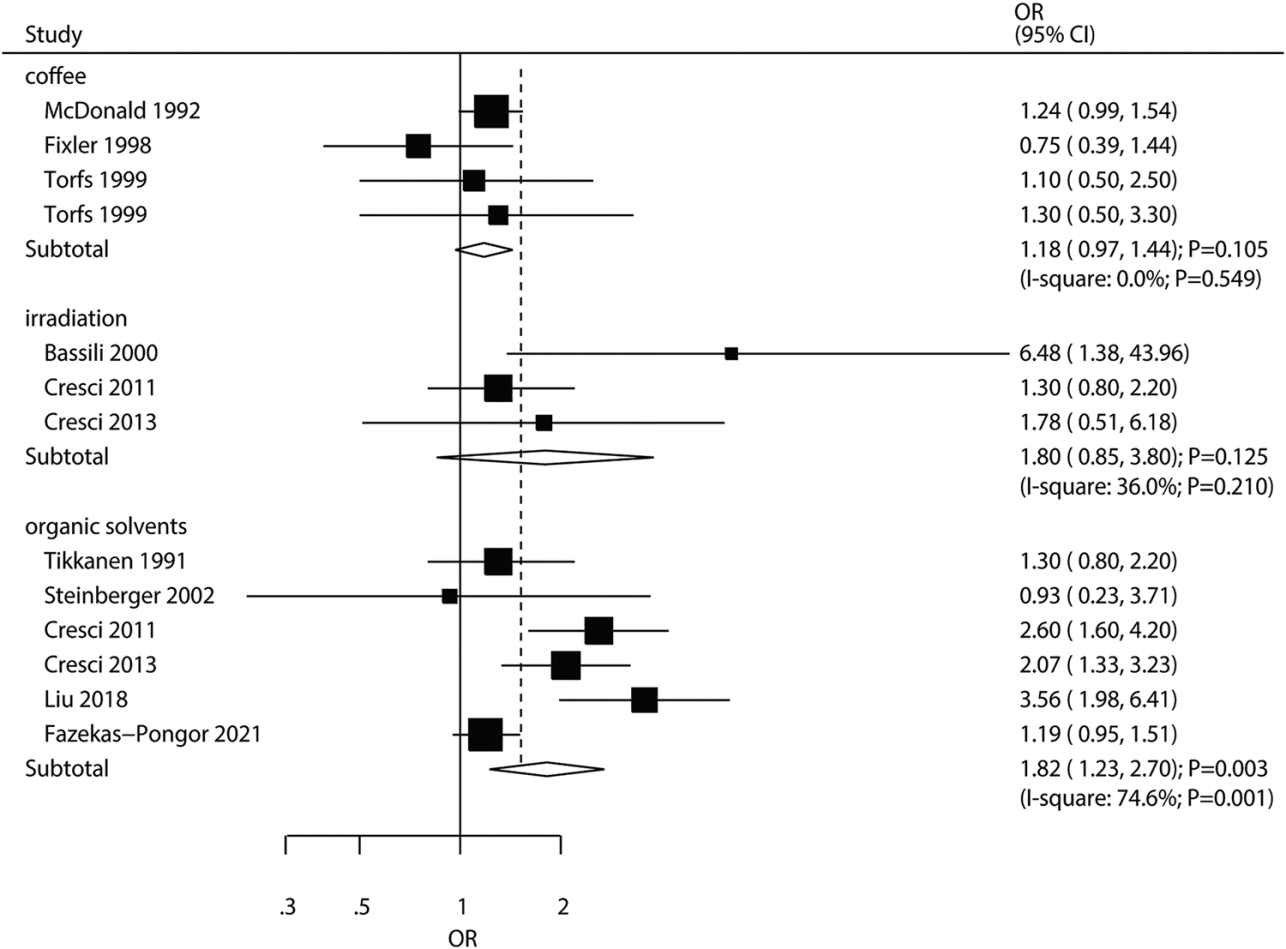
Associations of maternal coffee intake and exposure to irradiation or organic solvents with the risk of CHD in offspring

A total of three (four cohorts), two (three cohorts), and five (six cohorts) studies respectively evaluated the correlations of coffee consumption, irradiation and exposure to organic solvents with CHD risk in offspring. Exposure of pregnant women to organic solvents showed a significant association with elevated CHD risk (OR 1.82, 95% CI 1.23–2.70; *P* = 0.003), but not with maternal coffee consumption (OR 1.18, 95%CI 0.97–1.44; *P* = 0.105) and exposure to irradiation (OR 1.80, 95% CI 0.85–3.80; *P* = 0.125) (Fig. 7). There was significant heterogeneity among studies assessing exposure to organic solvents (*I*^2^ = 74.6%; *P* = 0.001). A moderate heterogeneity was detected across studies on irradiation (*I*^2^ = 36.0%; *P* = 0.210), and no evidence of heterogeneity was found among studies on maternal coffee intake (*I*^2^ = 0.0%; *P* = 0.549).

## Discussion

This meta-analysis focusing on observational trials evaluated the potential effects of maternal factors on CHD risk in offspring. A total of 182,290 CHD cases in offspring from 64 studies with various individual features were covered for a systematic review. As shown above, maternal obesity, smoking, diabetes and exposure to organic solvents were significantly associated with elevated CHD risk in children. Meanwhile, the correlations of maternal age, underweight in pregnancy, alcohol or coffee intake, and exposure to irradiation with CHD risk in offspring were not consistent. Furthermore, the associations between maternal factors and child CHD could be influenced by study design, reported outcomes and confounder-adjusted models.

The present meta-analysis demonstrated that maternal age was not associated with CHD risk in offspring. However, advanced maternal age could be linked with increased incidence of CHD in children after adjustment for certain confounding factors. It should be noted that many studies reported inconsistent data. Malik and colleagues reported that maternal age ≥ 35.0 (versus < 20.0 years) was associated with elevated CHD risk (Malik et al., 2008). In addition, Liu et al. indicated that maternal age ≥ 35.0 (versus 25.0–29.0 years) was related to an increased CHD risk in offspring (Liu et al., 2013). Furthermore, another study revealed an association between maternal age ≥ 36.0 (versus 15.0–29.0 years) and elevated CHD risk in children (Liu et al., 2018). This might be attributed to chromosomal abnormalities, such as trisomy 21 caused by meiotic nondisjunction errors as oocyte aged. Moreover, advanced maternal age could be linked with increased risk of multiple pregnancy-related complications, including spontaneous abortion, preeclampsia, gestational diabetes, fetal growth restriction and stillbirth (Cleary-Goldman et al., 2005; Jacobsson et al., 2004; Laopaiboon et al., 2014; Salem Yaniv et al., 2011; Stillbirth Collaborative Research Network Writing Group, 2011).

This meta-analysis demonstrated that maternal obesity, but not maternal underweight, had a significant association with CHD risk in offspring. These findings corroborated the findings in a previous meta-analysis (Zhu et al., 2018). These conclusions might be explained by the fact that maternal BMI was closely correlated with the intake of trans-fatty acids, and increased folate level could result in down-regulation of homocysteine (Davis et al., 2014). Obese pregnant women had lower folate and glutathione intake, which could lead to up-regulation of homocysteine level (Amirkhizi et al., 2014; Igosheva et al., 2010; Sanchez-Margalet et al., 2002; Vayá et al., 2012), thereby compromising the in-utero environment and impairing fetal development. Obstructive heart defect (OHD) is associated with variations in genes involved in homocysteine, folate and glutathione synthesis by the transsulfuration pathways. In addition, single nucleotide polymorphisms (SNPs) of multiple genes including genes encoding methylenetetrahydrofolate reductase, glutamate-cysteine ligase, betaine-homocysteine methyltransferase and DNA (cytosine-5-)-methyltransferase 3 beta were found to be related to increased OHD risk in females with obesity (Tang et al., 2015a). However, the association between maternal obesity and VSD risk was shown not to be statistically significant, which needed further investigation. Obese women might be susceptible to metabolic alterations, such as increased estrogen levels, hyperinsulinemia, hypertension, hyperglycemia and nutritional deficits, which increased the risk of congenital anomalies (Watkins et al., 2003). Abdominal adipose tissue accumulation was linked to the pathogenesis of diabetes, inflammation and metabolic disorders (Shaw & Carmichael, 2008). Abnormal glucose metabolism alone did not account for the elevated rates of congenital malformations in the offspring of obese women (Brite et al., 2014). It has been reported that obesity and diabetes can promote a variety of metabolic alterations, including abnormal lipid and carbohydrate metabolism, insulin resistance, altered activities of adipocyte hormones (Mills et al., 2010), disturbance in micronutrient metabolism, and elevated oxidative stress (Rankin et al., 2010). The intrauterine environment could be affected by nutritional and chemical changes during gestation. Elevated amounts of cytokines (such as interleukins, tumor necrosis factor-α, and monocyte chemotactic protein-1), leptin, procoagulant proteins and protein hormones were found in obese women, which increased the odds of maternal diseases and neonatal complications (Iessa & Bérard, 2015). In addition, obesity, insulin resistance and CHD such as myocardial contractile anomalies and cardiac hypertrophy in offspring could be caused by high fat diet exposure and maternal obesity (Dong et al., 2013).

This study showed that maternal alcohol intake was not associated with CHD risk in children. However, a potentially harmful impact of maternal alcohol intake on CHD risk was detected when pooling cohort studies. The findings of this study were consistent with those in previous meta-analysis (Sun et al., 2015). These conclusions might be explained by the fact that prenatal alcohol exposure could induce birth abnormalities, collectively referred to as fetal alcohol syndrome, and nearly 54% of live-born infants with this syndrome presented with cardiac anomalies (Karunamuni et al., 2014). In addition, maternal alcohol intake could modulate Wnt/β-catenin signaling, activating abnormal gene expressions in cardiogenesis (Serrano et al., 2010). As shown above, maternal smoking increased CHD risk in offspring, which was likely due to the teratogenic effect of smoking. Maternal cigarette exposure or even direct seminal fluid smoke exposure can cause genotoxicity (Gianicolo et al., 2010). Maternal smoking could affect the fetus due to the complex interaction of nicotine with fetal neurotransmitters (Paludetto et al., 2018). Fetal heart growth was hampered by abnormal DNA replication caused by toxins in cigarettes (Edwards & Gelb, 2016). Moreover, polymorphisms in maternal and fetal genes encoding excision repair cross-complementation group 1 (ERCC1), O-sialoglycoprotein endopeptidase, poly (ADP-ribose) polymerase 2 and ERCC5 were found to be associated with elevated risk of tobacco-associated CHD (Tang et al., 2015b). SNPs of genes encoding the glutathione-S-transferase (GST) family proteins that could alleviate oxidative stress, could also increase the risk of smoking-associated CHD and affect the expressions of GSTA4 and glutamate-cysteine ligase, the rate-limiting enzyme of glutathione synthesis contributing to DNA methylation and transsulfuration in the fetus. Additionally, SNPs in genes encoding replication factor c subunit 1 (fetal and maternal) and nitric oxide synthase 3 (fetal) involved in DNA synthesis were also shown to be linked with CHD risk (Edwards & Gelb, 2016). As demonstrated earlier, maternal diabetes showed a significant association with elevated CHD risk in children, corroborating previously reported findings (Hoang et al., 2017). This conclusion was likely due to the potential differences in the impacts of gestational and pre-gestational diabetes on CHD in offspring (Holing et al., 1998; Ray et al., 2001). Additionally, the prediabetic state in pregnant women could affect the occurrence and development of CHD in offspring, although factors related to prediabetes were not mentioned (Loeken, 2014; Lupo et al., 2012). Cardiac malformation in diabetic embryopathy deserves further investigation. However, the prevailing hypothesis is that excess glucose has a teratogenic effect on the developing heart. Glucose may indirectly exert this effect through the signaling pathways that control insulin sensitivity, which is the key regulator of embryogenesis and early embryonic development. Epigenetic changes resulting from histone acetylation and specific microRNA expressions affected by glucose or inherited genetic variations from diabetic women are additional possible causes of CHD (Øyen et al., 2016). Multifactorial processes seem to be associated with CHD risk in the offspring of obese women. Oxidative stress, Wnt signaling, nitric oxide and Notch signaling, the TGF-β pathway and the Hif1α pathway, which play critical roles in the early stages of cardiac development, have been implicated in diabetic embryopathy. Genetic analyses revealed the roles of ligands and receptors in cell signaling pathways (JAG1, NOTCH1 and NOTCH2), transcription factors (GATA4, TBX5 and NKX2.5), laterality pathway-related proteins (NODAL, LEFTY and CITED2) and structural proteins (ACTC1, MYH6, MYH7 and MYH11) in CHD occurrence and development (Basu & Garg, 2018). Moreover, the above results indicated that exposure of pregnant women to organic solvents was associated with CHD risk in offspring, while maternal coffee intake and irradiation did not show this relationship. Organic solvents, such as cleaning fluids, stain removers, paint thinners and nail polish removers, have been implicated to be associated with *MTHFR* 677 CC genotype. Meanwhile, transforming growth factor beta (TGF-β) receptor type 1 (TGFBR1) and TGFBR2 gene alterations are linked to patent ductus arteriosus (Nicoll, 2018). However, only a few studies have investigated the influence of these factors in pregnant women.

Maternal predisposing factors for CHD may expand the scope of CHD risk assessment in offspring, ultimately helping to reduce CHD incidence in children. Given that congenital heart defects in fetuses can lead to early miscarriage, the results of this study may provide better pregnancy management. In this meta-analysis, substantial heterogeneity was found across the studies assessing the influence of maternal age, alcohol intake, smoking, diabetes, coffee, irradiation, and exposure to organic solvents on CHD in offspring. Subgroup analysis revealed that study design, CHD type (ASD versus VSD) and confounders might be the sources of heterogeneity, as heterogeneity could be reduced when switching these subsets.

The limitations of the current study should be highlighted. First, most included studies were designed as an observational case–control trial, and uncontrolled selection and recall bias were inevitable. Second, different cutoff values and adjusted confounders were adopted by various studies, which might influence the estimate of CHD risk in offspring. Third, the included studies used various reference groups to investigate maternal factors, which might affect the pooled results. Fourth, this meta-analysis assessed reported studies, and publication bias was unavoidable. Fifth, the association analyses were based on pooled data, and various parameters in individual studies could not be comprehensively analyzed. Sixth, a small number of studies might have low statistical power to detect a difference between the CHD and non-CHD groups (for coffee and irradiation respectively). Substantial heterogeneity was detected across the studies on the influence of maternal exposure to organic solvents on child CHD. Furthermore, certain studies were conducted among infants with known chromosomal/genetic or maternal drug defects. These characteristic might affect other maternal factors and the outcome of CHD.

## Conclusion

In conclusion, the current study indicated that maternal obesity, smoking, diabetes and exposure to organic solvents were significantly associated with elevated CHD risk in offspring. However, maternal age, underweight in pregnancy, alcohol and coffee intake and exposure to irradiation were not linked with offspring CHD risk. However, large prospective trials are warranted to confirm these findings.

## Supplementary Information

Below is the link to the electronic supplementary material.

Supplementary file1 (DOC 1208 KB)

Supplementary file2 (DOC 649 KB)

## Data Availability

All data generated or analyzed during this study are included in this article and its supplementary material files. Further enquiries can be directed to the corresponding author.
