# Supplementary material for: Association Between Maternal Factors and Risk of Congenital Heart Disease in Offspring: A Systematic Review and Meta-Analysis
Source: Matern Child Health J. 2022 Nov 7;27(1):29–48. doi: 10.1007/s10995-022-03538-8 (PMC9867685; doi:10.1007/s10995-022-03538-8)
Supplement: Supplementary file 2 — Supplementary file2 (DOC 649 KB) [file 10995_2022_3538_MOESM2_ESM.doc]

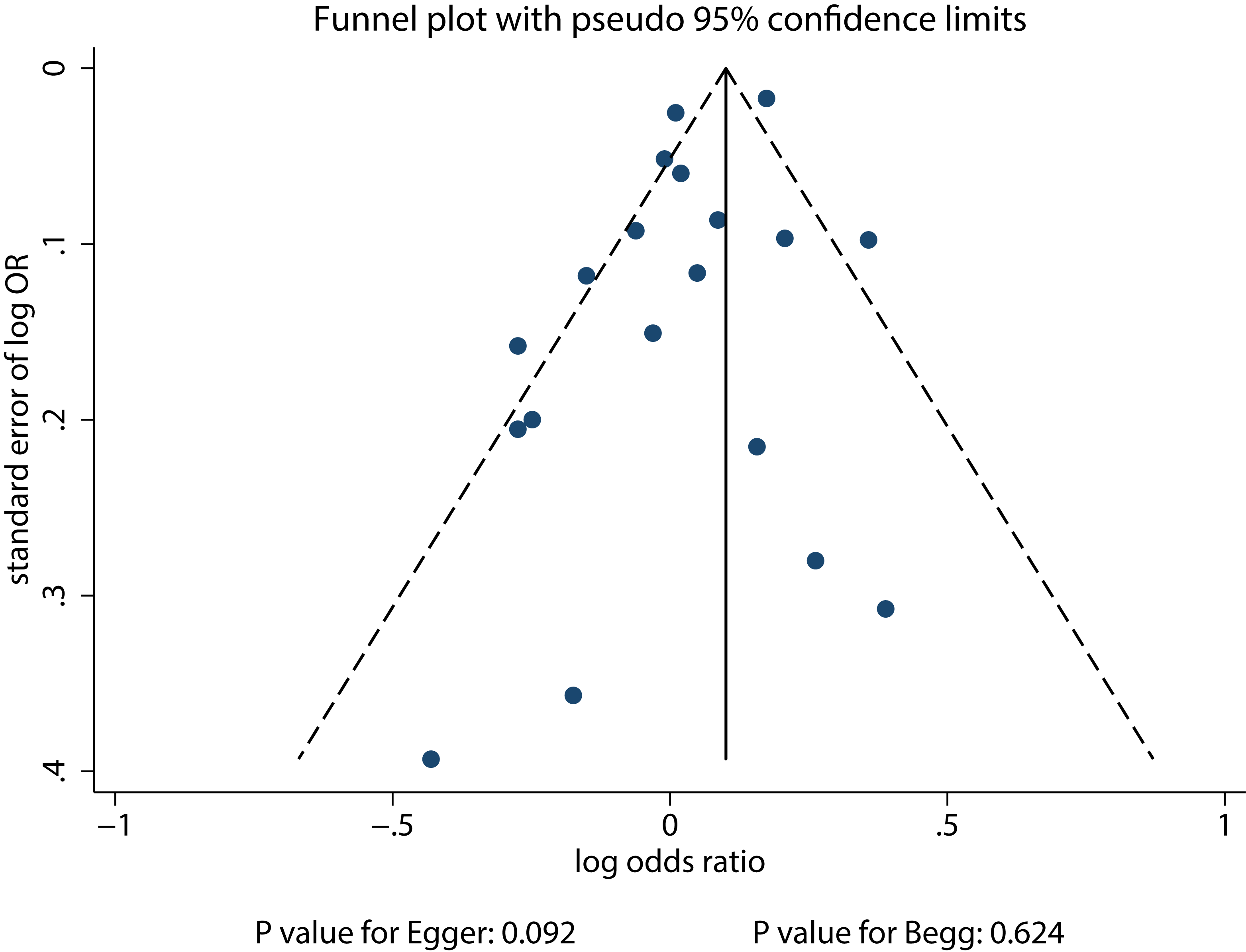


**Figure S1.** Funnel plot for the association of maternal age with the risk of CHD in offspring


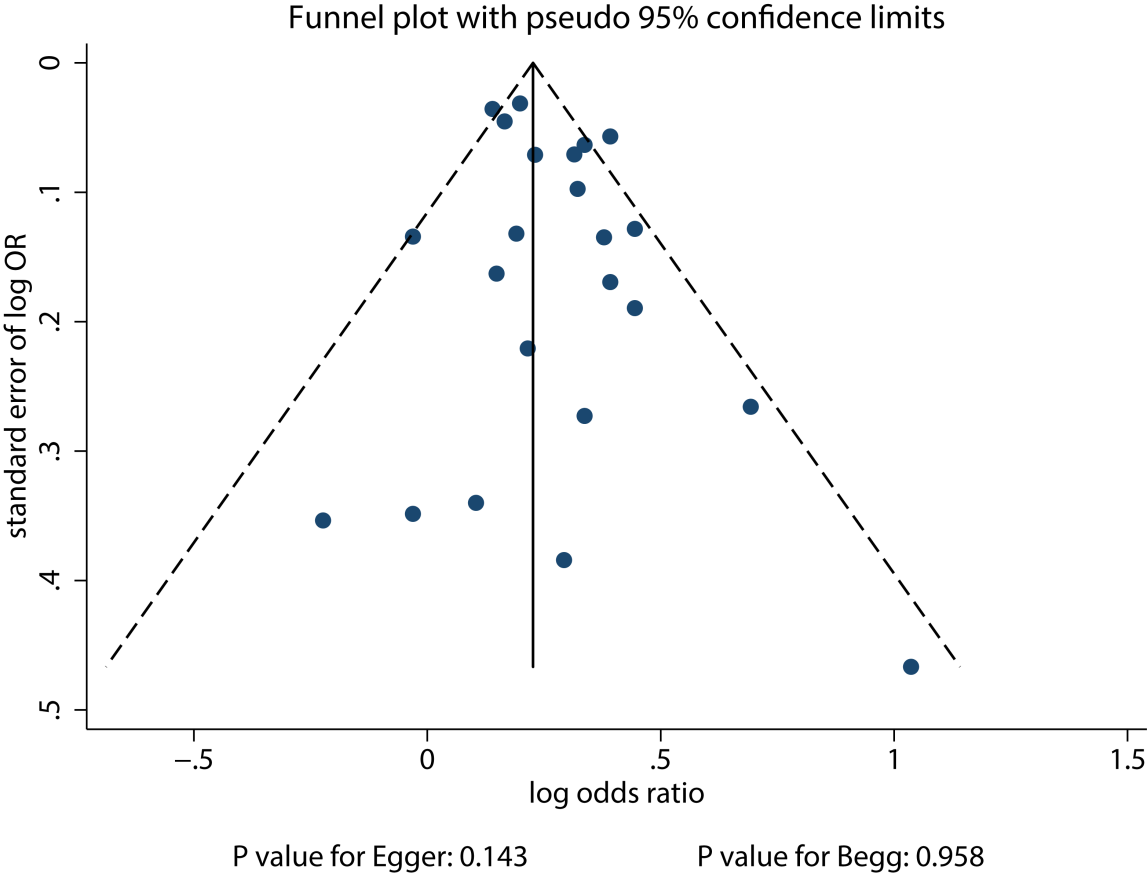


**Figure S2.** Funnel plot for the association of maternal obesity with the risk of CHD in offspring


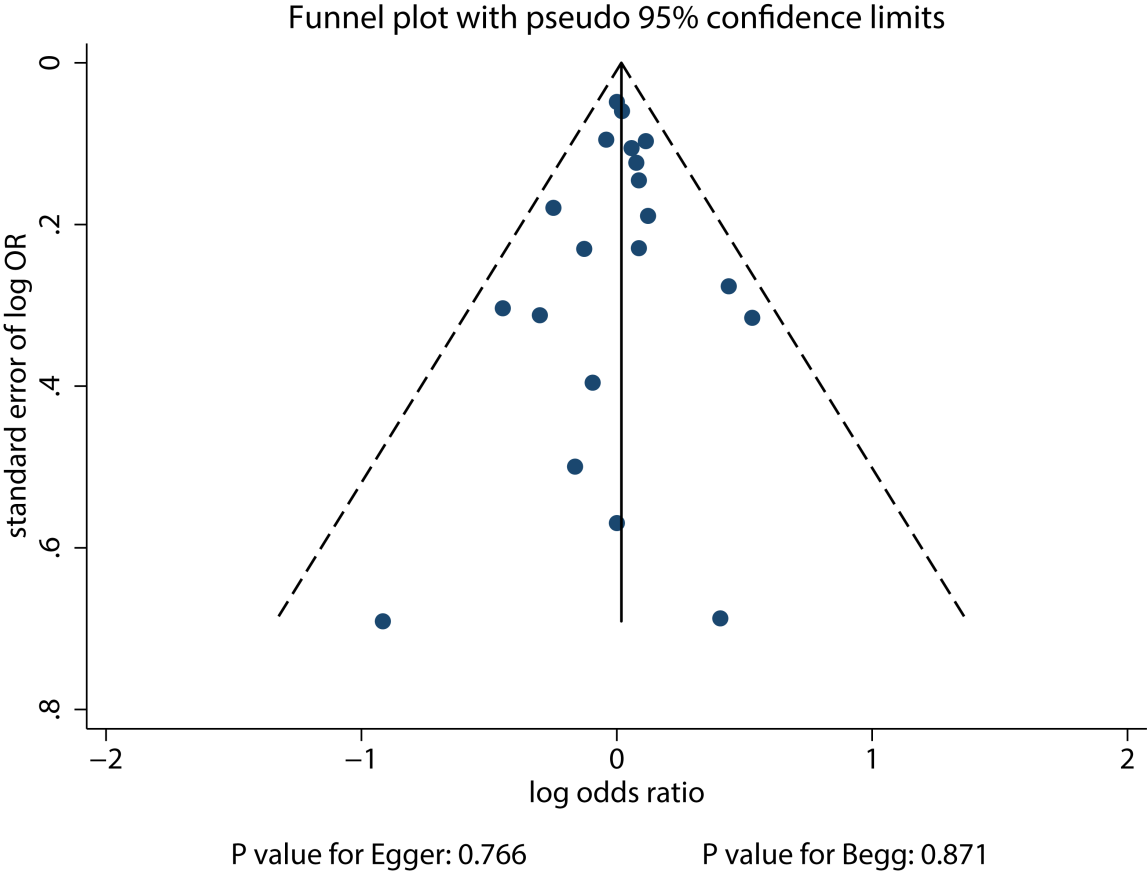


**Figure S3.** Funnel plot for the association of maternal underweight with the risk of CHD in offspring


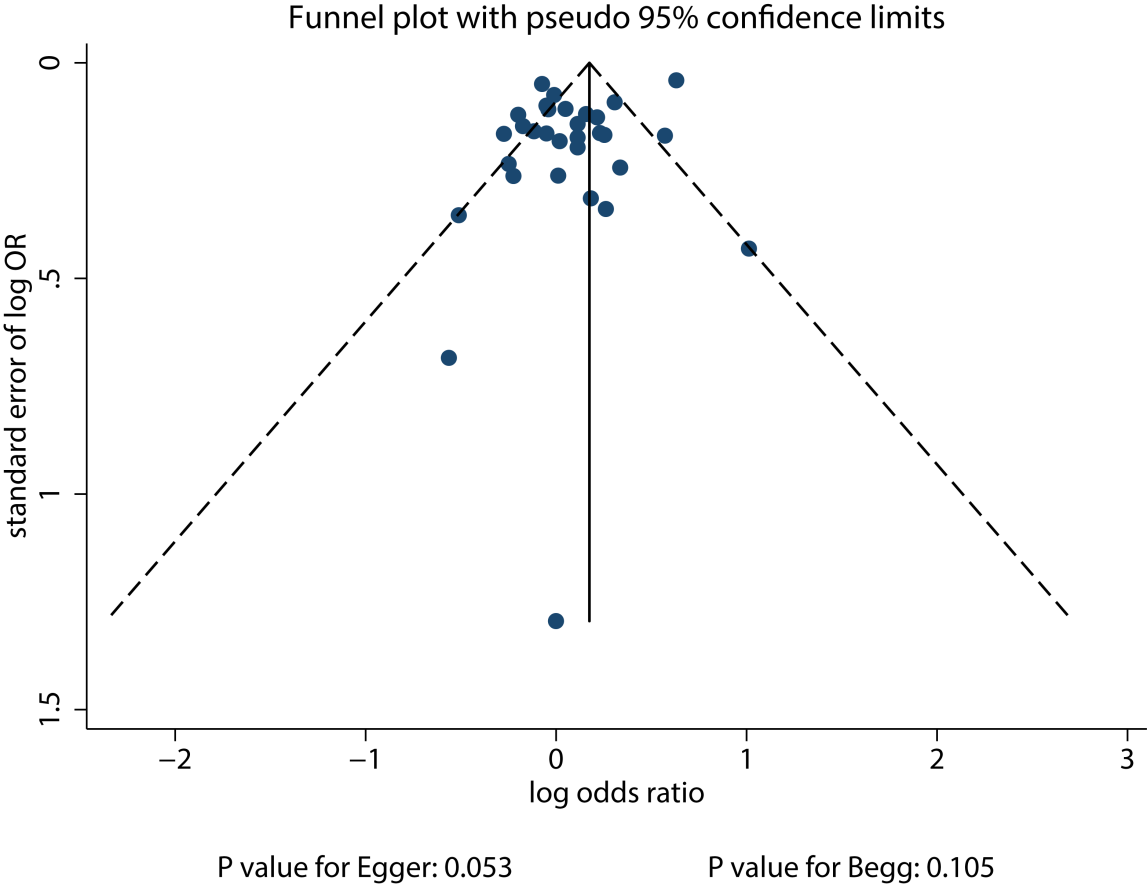


**Figure S4.** Funnel plot for the association of maternal alcohol intake with the risk of CHD in offspring


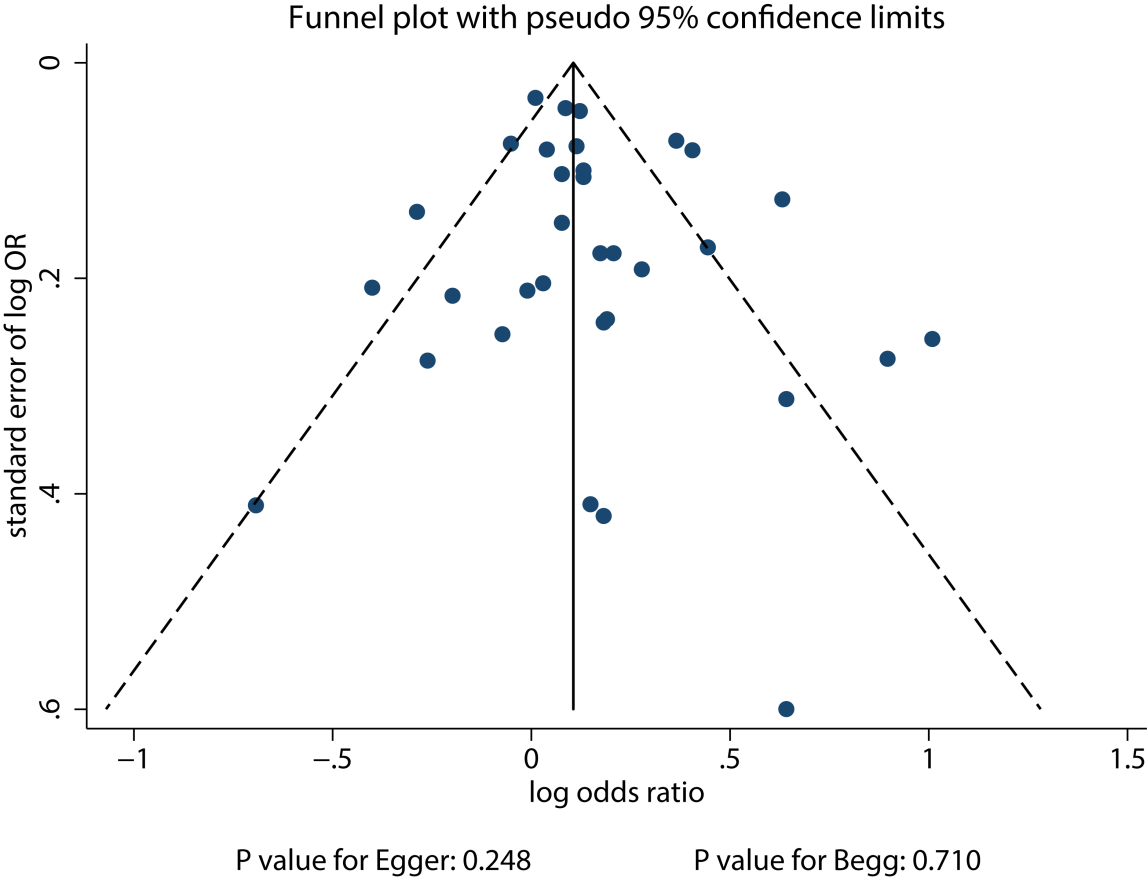


**Figure S5.** Funnel plot for the association of maternal smoking with the risk of CHD in offspring


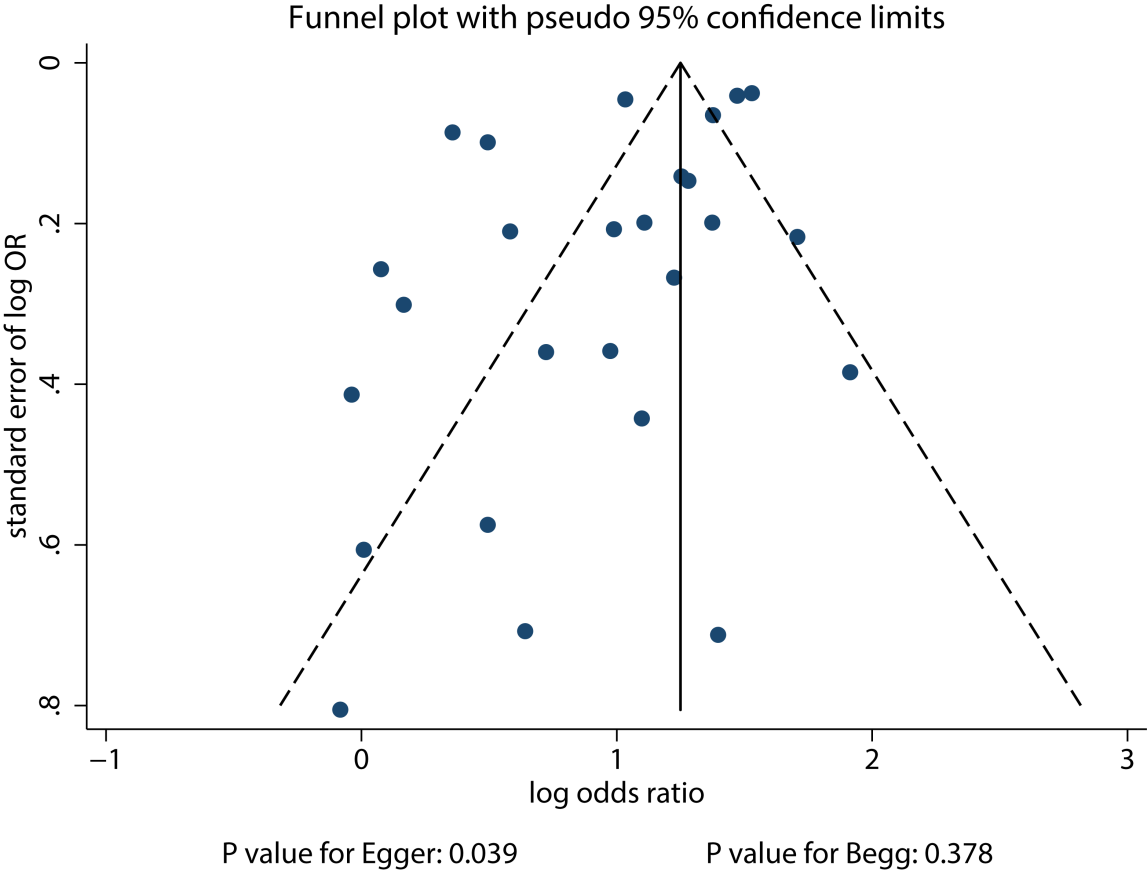


**Figure S6.** Funnel plot for the association of maternal diabetes with the risk of CHD in offspring
